# A Good Death? Report of the Second Newcastle Meeting on Laboratory Animal Euthanasia

**DOI:** 10.3390/ani6090050

**Published:** 2016-08-23

**Authors:** Penny Hawkins, Mark J. Prescott, Larry Carbone, Ngaire Dennison, Craig Johnson, I. Joanna Makowska, Nicole Marquardt, Gareth Readman, Daniel M. Weary, Huw D. R. Golledge

**Affiliations:** 1 Research Animals Department, Royal Society for the Prevention of Cruelty to Animals (RSPCA), Wilberforce Way, Southwater, Horsham, West Sussex RH13 9RS, UK; penny.hawkins@rspca.org.uk; 2 National Centre for the Replacement, Refinement and Reduction of Animals in Research (NC3Rs), Gibbs Building, 215 Euston Road, London NW1 2BE, UK; 3 Laboratory Animal Resource Center, University of California, San Francisco, CA 94143, USA; larry.carbone@ucsf.edu; 4 Animals in Science Regulation Unit, Home Office Science, 1st Floor Peel NE, 2 Marsham Street, London SW1P 4DF, UK; n.dennison@dundee.ac.uk; 5 Institute of Veterinary, Animal and Biomedical Sciences, Private Bag 11 222, Massey University, Palmerston North 4442, New Zealand; c.b.johnson@massey.ac.nz; 6 Animal Welfare Program, University of British Colombia, 2357 Main Mall, Vancouver, BC V6T 1Z4, Canada; makowska@interchange.ubc.ca (I.J.M.); dan.weary@ubc.ca (D.M.W.); 7 Institute of Pharmacology and Toxicology, Freie Universität Berlin, Koserstraße 20, Berlin 14195, Germany; marquardt@berliner-fortbildungen.de; 8 School of Biology, University of Plymouth, Smeaton 008D, Drakes Circus, Plymouth PL4 8AA, UK; gareth.readman@plymouth.ac.uk; 9 Universities Federation for Animal Welfare (UFAW), The Old School, Brewhouse Hill, Wheathampstead, Hertfordshire AL4 8AN, UK; golledge@ufaw.org.uk

**Keywords:** animal welfare, carbon dioxide, euthanasia, humane killing, mouse, rat, refinement, 3Rs, zebrafish

## Abstract

**Simple Summary:**

Millions of laboratory animals are killed each year worldwide. However, there is a lack of consensus regarding what methods of killing are humane for many species and stages of development. This report summarises research findings and discussions from an international meeting of experts and stakeholders, with recommendations to inform good practice for humane killing of mice, rats and zebrafish. It provides additional guidance and perspectives for researchers designing projects that involve euthanasing animals, researchers studying aspects of humane killing, euthanasia device manufacturers, regulators, and institutional ethics or animal care and use committees that wish to review local practice.

**Abstract:**

Millions of laboratory animals are killed each year worldwide. There is an ethical, and in many countries also a legal, imperative to ensure those deaths cause minimal suffering. However, there is a lack of consensus regarding what methods of killing are humane for many species and stages of development. In 2013, an international group of researchers and stakeholders met at Newcastle University, United Kingdom to discuss the latest research and which methods could currently be considered most humane for the most commonly used laboratory species (mice, rats and zebrafish). They also discussed factors to consider when making decisions about appropriate techniques for particular species and projects, and priorities for further research. This report summarises the research findings and discussions, with recommendations to help inform good practice for humane killing.

## 1. Introduction

The vast majority of animals used in research and testing worldwide are killed, either because their tissues or organs are required at the end of a study, because a **humane** **endpoint** has been reached, or because they are surplus to requirements (e.g., due to overbreeding or if they do not have a desired genotype). There are ethical and legal reasons for ensuring that laboratory animals are killed as humanely as possible. However, there is debate regarding the relative **humaneness** of, and best practice for, a number of commonly used techniques, such as carbon dioxide (CO_2_).

In recognition of this, a meeting on carbon dioxide euthanasia of laboratory animals was held at the Newcastle University in 2006, with support from the UK’s National Centre for the Replacement, Refinement and Reduction of Animals in Research (NC3Rs) and Laboratory Animals Limited. This initial meeting aimed to bring together scientists who had researched the use of CO_2_ for killing laboratory rats and mice, to help inform best practice for CO_2_ **euthanasia**. Additional aims were to identify what research was needed to further identify and evaluate welfare issues associated with the use of CO_2_, and to consider whether any preferable alternatives were currently available.

The initial Newcastle meeting report [1] set out the majority view of the meeting participants. It identified animal welfare problems with CO_2_ killing and made recommendations for good practice, but could not recommend any other **inhaled** **agents** apart from volatile anaesthetics, with the caveat that the **aversiveness** of these can vary. A list of recommendations was made for future research, to identify good practice and evaluate potential alternatives to CO_2_.

The 2006 report raised the profile of the debate regarding CO_2_, prompting a number of establishments to review their CO_2_ administration protocols, or to induce general anaesthesia with an inhaled anaesthetic agent before switching to CO_2_. A further outcome of the meeting was the announcement by the NC3Rs of a call for applications for a Strategic Award aimed at answering some of the identified research needs, which was ultimately made to Dr Huw Golledge of Newcastle University. A component of the NC3Rs award included funding to run a second meeting on laboratory animal euthanasia, to provide an update on progress, which is the subject of this report.

Since the 2006 meeting, many regulations and/or guidelines governing the use of humane killing techniques for laboratory animals have been revised, including those issued by the European Union [2] and its Member States, the American Veterinary Medical Association [3] and the Canadian Council on Animal Care [4]. Some key changes include:

- the use of CO_2_ should only be in a rising concentration [2,3];
- the recommendation that CO_2_ should not be used as a sole killing agent on conscious animals [4];
- addition of the use of inert gases as an “acceptable” technique for rodents [2];
- a presumption that decapitation is only acceptable when no other method is possible [2];
- the opposite presumption that the only welfare concern with decapitation and cervical dislocation is the skill of the person [3].

The impact of these policy changes on animal welfare is variable. For example, as reviewed below, administering CO_2_ in a rising concentration (as opposed to pre-fill) will avoid pain but may increase the duration of fear and distress relative to the pre-fill method.

The overall aim of the second Newcastle Meeting, held on 9 August 2013, was to provide a forum to discuss recent research and current practice on euthanasia, so as to inform debate and policy on humane killing (for the meeting programme, see Supplementary Materials Table S1). The specific aims were to:

- review key areas of recent research into humane killing;
- consider how this could be interpreted and incorporated into practice;
- expand the remit beyond CO_2_, to include recent studies that have evaluated the humaneness of some physical methods for killing rodents and the use of anaesthetics and inert gases for killing rodents;
- include zebrafish as well as mice and rats, to encompass the three most commonly used laboratory species;
- discuss special considerations when killing **neonatal** animals;
- incorporate extended discussion sessions to explore ethical issues and criteria for judging the **humaneness** of different techniques and devices.

**Figure 1:**
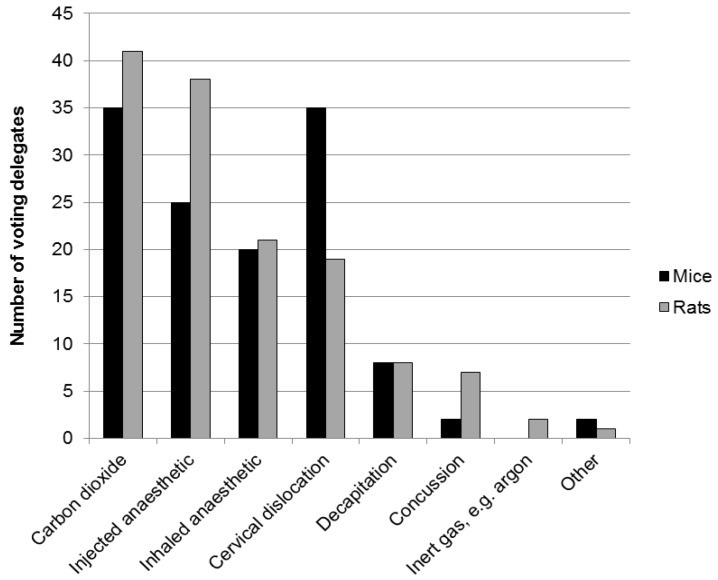
Meeting participants’ responses to the questions: “Which methods do you use to kill mice/rats?”.

**Figure 2:**
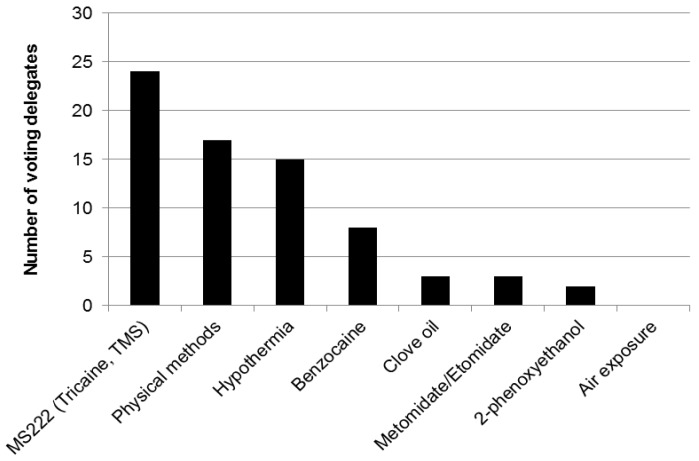
Meeting participants’ responses to the question: “Which methods do you use to kill fish?”.

Demographic information, participants’ views and information on current practices were obtained using an interactive, electronic polling system (Turning Technologies, LLC). The audience of 90 participants included researchers, veterinarians, facility managers, animal technologists, research regulators, and staff of animal welfare and 3Rs organisations. Most participants worked within academic institutions. Two thirds were based in the UK and one sixth in other EU member states, with the remainder from the USA, Canada, Japan and New Zealand. Four fifths of the audience were directly involved in the euthanasia of animals used in research; mostly rats and mice but also fish and birds. Participants directly involved in euthanasia reported the use of a variety of techniques for mice and rats (Figure 1) and fish (Figure 2).

Section 2 of this report summarises the speakers’ presentations and sets out the majority views of the meeting participants on some selected topics, which were obtained using the interactive electronic polling system during the discussion sessions. A summary of the main points and areas for further research is given in Section 3 and conclusions and recommendations are set out in Section 4. The overall aim is to provide information and points to consider for a range of audiences worldwide, including researchers designing projects that may involve euthanising animals, researchers studying aspects of humane killing (e.g., evaluating the physiology of the killing process or the humaneness of different techniques), veterinarians, animal technologists, euthanasia device manufacturers, regulators, and institutes that wish to review local practice and training for humane killing (e.g., via review by the ethics or animal care and use committee).

## 2. Summary of the Meeting

The welfare implications of any humane killing technique will depend upon the individual animal’s species, strain and stage of development. The meeting focused on inhaled agents such as anaesthetics and CO_2_, decapitation and cervical dislocation in mice and rats, and addition of agents to tank water in zebrafish, as these techniques and species account for the majority of killing in the laboratory.

Euthanasia techniques ultimately cause death by three basic mechanisms: (i) direct depression of neuronal activity necessary for life function; (ii) **hypoxia**; or (iii) physical disruption of brain activity. By definition, welfare concerns can occur only during the period of consciousness; what happens between the complete loss of consciousness and death should not be an issue because an animal cannot suffer during this period. However, for some methods there may be a period of full or partial consciousness for some time after the initial insult has occurred.

The animal’s experience between the commencement of the killing technique, and the final loss of consciousness, is only one consideration when determining the relative humaneness of a euthanasia protocol. Humane killing is a *process* during which the animal may experience a number of stressful, distressing or painful events, depending on the technique, including capture, handling, restraint, separation from cage-mates, exposure to unfamiliar conspecifics, transport, relocation to a different enclosure [5,6,7] and pain (e.g., due to injection, or the chemical properties of the euthanasia agent). The potential for recovery from the technique is also an important criterion for determining the humaneness of some methods. Although all currently available techniques will inevitably involve some degree of pain and/or distress, due consideration of all of the above elements should help to determine the most humane method. The sub-sections below should be read in this context, although the focus is on reducing suffering around application of the technique.

### 2.1. Inhaled Agents in Rodents

Most laboratory rodents are killed using inhalation techniques. European Directive 2010/63/EU permits two inhalation techniques for rodents: a gradually rising concentration of CO_2_ or inert gases (argon or nitrogen) (The use of inert gases for killing laboratory animals is not permitted in the UK, because they are considered to be aversive (argon) or there is insufficient evidence that they are humane (nitrogen)). In addition, inhaled anaesthetics may be used to anaesthetise animals before switching to CO_2_ to complete the euthanasia process. The AVMA euthanasia guidelines also state that inhaled anaesthetics or a gradual fill of CO_2_ (the latter with or without premedication with inhaled anaesthetics) are acceptable for rodents, but lists nitrogen and argon amongst unacceptable methods, unless animals are anaesthetised [3].

#### 2.1.1. **Aversion** to CO_2_ and Inhaled Anaesthetics

Of the many studies examining responses of rodents to CO_2_, all show that the agent is **aversive**. Given that rats and mice consistently avoid CO_2_ when they are able to, they are likely to experience distress if they cannot escape, as is the case during euthanasia. At the meeting, **Joanna** **Makowska** reviewed the results from a wide cross-section of studies that consistently demonstrate that mice and rats show aversion to CO_2_ even if this involves foregoing a desirable food reward or exposing themselves to another aversive stimulus.

The **approach-avoidance** paradigm has been used to evaluate mouse and rat aversion to CO_2_, argon and inhaled anaesthetics. Mice and rats exposed to a rising concentration of CO_2_ leave a test chamber at the cost of abandoning food rewards when levels rise to approximately 12%–18% [8,9,10]. With O_2_ supplementation, rats will stay until the concentration reaches around 21% CO_2_ [11]; however, as noted by the study authors, this reduction is not enough to warrant recommending the use of CO_2_ and O_2_ mixtures for killing rats. Even animals that have been deprived of food for 24 h (and hence are highly motivated to access the food reward) will escape the chamber when CO_2_ reaches approximately 12%–18% [12]. This research supports earlier work demonstrating that CO_2_ is aversive [1].

One potential criticism of the approach-avoidance paradigm is that CO_2_ may affect taste receptors, making the sweet food reward less attractive. **Aversion-avoidance** testing cannot be subjected to this criticism as it compares motivation to avoid the gas with motivation to avoid an open, brightly lit chamber (known from a large body of work to be fear inducing in rodents). As with the approach-avoidance tests, mice and rats tested using the aversion-avoidance paradigm never stay in a chamber filling with CO_2_ and instead escape to the brightly lit open chamber they otherwise avoid [13,14]. Thus across a large number of peer-reviewed studies, using a range of species, strains and testing methods, we see a highly consistent aversion response to concentrations of CO_2_ much lower than are required for anaesthesia.

Evidence also indicates that exposure to volatile anaesthetics is a refinement over exposure to CO_2_. In approach-avoidance testing, most naïve rodents choose to remain in a chamber (with a food reward) filling with isoflurane, sevoflurane or halothane until they are ataxic, and some rodents never leave [15,16,17]. Similarly, in aversion-avoidance testing, most naïve rodents tested with these agents remain in the dark chamber until they lose consciousness, rather than escaping to the brightly lit compartment [13,14,15].

However, all available evidence indicates that repeated exposure to the volatile anaesthetics is more aversive than the initial exposure. A series of studies have shown that mice and rats are more likely to escape the test chamber, and to do so more quickly, on second and subsequent exposures to volatile anaesthetics [13,14,15]. In contrast, aversion to CO_2_ does not significantly increase on subsequent exposure [14]. This could be a “floor effect”, in which aversion to CO_2_ is already maximal and cannot increase further. Alternatively, it could reflect the innate nature of aversion to CO_2_, a naturally occurring substance to which rodents may have evolved to be averse, as opposed to isoflurane, a synthetic agent with a pungent odour (to humans, at least) which could serve as a potent unconditioned stimulus. The similarity in odour of isoflurane and sevoflurane may explain the generalization of the conditioned response to isoflurane to sevoflurane.

So, although the volatile agents can be an important refinement over CO_2_ (and **anoxia**), these agents cannot be considered ideal; some animals choose to escape the test chamber when exposed to these agents within both approach-avoidance and aversion-avoidance paradigms. The available evidence indicates that isoflurane and sevoflurane appear to be equally aversive to mice and rats, and that halothane is more aversive [15,16]. Most of the literature relates to volatile anaesthetics delivered using a precision vaporiser, but these agents can also be administered using the so-called “drop method” whereby the agent is delivered as a liquid (typically absorbed onto a swab) and vaporised rapidly within the chamber, allowing a much higher concentration to be achieved than the maximum output from a vaporiser (typically 5% for isoflurane). In this case, it may be possible to kill animals in a relatively short period of time; however, mice exposed to a rapidly rising concentration of isoflurane using the “drop technique” showed a higher level of aversion than those exposed using a vaporiser [13].

On the basis of published aversion studies available at the time (2006), the previous Newcastle meeting recommended that inhalant anaesthetics be used to render animals unconscious before switching to CO_2_. (Because of their wide safety margins, it is time consuming to kill animals with isoflurane [18] or halothane, so a secondary method is sometimes preferred for the sake of efficiency and safety). However, subsequent aversion studies have indicated that this may not necessarily be a refinement if animals have previously been exposed to inhalant anaesthetics (see below).

It is important to note that the time from unconsciousness to death has no bearing on animal welfare; as emphasised above, welfare concerns cease once the animal has been rendered unconscious. However, there can be serious welfare concerns, if poor technique results in some animals recovering consciousness before the secondary method takes effect. A study by Moody et al. [19], using an adapted chamber that accommodates an investigator’s hand yet is still airtight, has enabled righting and pedal withdrawal reflexes to be monitored in mice. This study showed that, with a flow rate of 17% chamber volume per minute of 5% isoflurane in O_2_, mice become recumbent after approximately 35 s from the beginning of exposure. However, the righting reflex is present until a mean of 45 s has elapsed, and the pedal withdrawal reflex is not lost until a mean of 75 s. On this basis and with a cautious safety margin (mean + 3 S.D.), when using the above administration protocol and CO_2_ exposure as the secondary method, CO_2_ should not be applied until at least 80 s after the onset of recumbency to prevent recovery of consciousness, which has been seen when switching from isoflurane to high flow rates of CO_2_ immediately after loss of consciousness [20].

To summarise findings in this field to date, in aversion-avoidance tests both mice and rats will always choose exposure to light over exposure to CO_2_, but more than 50% of mice and rats will tolerate *first* exposure to an inhalant anaesthetic until they become recumbent. However, both species also show evidence of learned aversion to inhaled anaesthetic agents. The reasons for this are as yet unknown, and it was suggested during discussion at the meeting that it may be due to memories of unpleasant side effects when waking from the first exposure; but it was also suggested that nausea or the excitation response during induction may contribute to aversion.

Whatever the cause, learned aversion to inhaled anaesthetic agents is likely to present animal welfare issues when mice and rats are repeatedly anaesthetised, for example during longitudinal studies that involve serially imaging animals. It is also likely that the experience of euthanasia using an inhalant anaesthetic will depend upon whether an animal has been anaesthetised before (and potentially the interval between the anaesthetics, i.e., whether the aversion is reduced or extinguished, which has not been investigated). The way in which animals appear to generalise between isoflurane, halothane and sevoflurane suggests that using a different but chemically-related anaesthetic agent for euthanasia will not solve the problem. All commonly used inhaled anaesthetics belong to the same chemical family of halogenated compounds and may therefore also elicit generalised responses. There was some discussion at the meeting as to whether it might be more humane to use CO_2_ to kill animals if they have been exposed to inhaled anaesthetics previously, but in the aversion-avoidance tests published to date, the percentage of mice and rats staying in the dark compartment with the anaesthetic never fell to zero, as it did for CO_2_. Furthermore, animals previously exposed to isoflurane did not show greater aversion (on average) to isoflurane than they did to CO_2_. As such, current evidence suggests that there is no welfare advantage to killing animals with CO_2_ if they have been previously exposed to isoflurane. Even in the worst case scenario so far tested isoflurane was only *as* aversive as CO_2_.

Results to date do suggest that episodes of general anaesthesia are not independent events for mice and rats, and therefore the number of techniques animals undergo that require general anaesthesia with inhaled anaesthetic agents (e.g., surgery or imaging) should be minimised and factored into harm-benefit analyses. More studies are required to elucidate the magnitude of aversion in animals exposed to isoflurane more than twice, and the effect on the magnitude of aversion of longer or short intervals between anaesthesia.

Aversion to an agent is generally interpreted as indicating that forced exposure (beyond the point of aversion) is distressing, but some caveats were discussed by **Huw Golledge**. Behavioural signs of apparent distress during forced exposure can be difficult to interpret; for example, some behavioural responses during exposure to volatile anaesthetics (such as increased locomotor activity, e.g., running) may not be consciously perceived by the animal as distressing. Similarly, a freezing response to a fear-inducing agent (such as CO_2_) can be mistakenly interpreted as a lack of distress. Correlating these specific behaviours with physiological parameters can help to determine their value as indicators of adverse physiological stress or distress [21]. Even some avoidance responses may be difficult to interpret; for instance, a wide range of taxa avoid exposure to high concentrations of CO_2_, even the nematode worm *C. elegans* [22], so an avoidance response *per se* may not be strong evidence for a consciously felt negative effect. Of course, the more sophisticated testing methods (aversion-avoidance and approach-avoidance), where animals must trade off one motivation for another, cannot be so easily dismissed. However, even in these cases results could be confounded if, for instance, the test agents affected the animal’s motivation to retrieve a reward as opposed to causing distress. Furthermore, physiological indicators of stress can be elevated by **eustress**, or can be elevated in unconscious animals, and therefore cannot be unequivocally linked to negative emotional states. Ideally, therefore, physiological and behavioural measures should be combined. Evidence that an inhaled agent is capable of causing psychological distress (and would cause distress if exposure was inescapable) is significantly strengthened where behaviours which could indicate distress is observed at concentrations that have been shown to induce physiological signs of stress, and where the agent can be shown to cause an animal to either forego a reward, or expose themselves to a stressful environment, in order to avoid the agent. Where aversion-avoidance or approach-avoidance studies result in significantly lower signs of aversion, there is good reason to argue that the behavioural signs of excitation may not be representative of distress. In such cases, any observed signs of physiological stress could represent stress that is not correlated with a negative affective state, e.g., that arising from increased locomotor activity.

A behavioural testing approach that has been successfully applied in zebrafish (see Section 2.4) is the **conditioned place aversion** paradigm. This test relies on the animal’s memory of the effects of exposure to the agent. The animal makes an association between the **affective state** induced by the agent (how they feel when they are exposed to the chemical, gas or anaesthetic vapour) and environmental cues. That is, there is a **Pavlovian association** between the agent (the unconditioned stimulus) and the environment (the conditioned stimulus). The choices that an animal makes in the absence of the test agent can thus provide insight into each animal’s memory of their subjective state during exposure to the agent. Development of conditioned place aversion therefore relies on the animal’s emotional experiences and is not subject to the same confounds as other behavioural approaches discussed in the preceding paragraph.

The **conditioned place preference**/aversion paradigm also employs protocols for exposing the animals to the test agent, when they are being conditioned, that are tailored to match conditions during the euthanasia process. Rats are handled immediately before the procedure, the fill rates are identical to those used in practice, and animals are conditioned for 90 s, until they are on the brink of losing consciousness. Furthermore, during conditioning exposures the animal is confined to a chamber and exposed to the agent, unlike approach-avoidance or aversion-avoidance studies where the animal is free to terminate exposure by leaving the chamber at any time. The experience of animals undergoing conditioning therefore mimics much more closely the experience of animals undergoing euthanasia.

**Figure 3:**
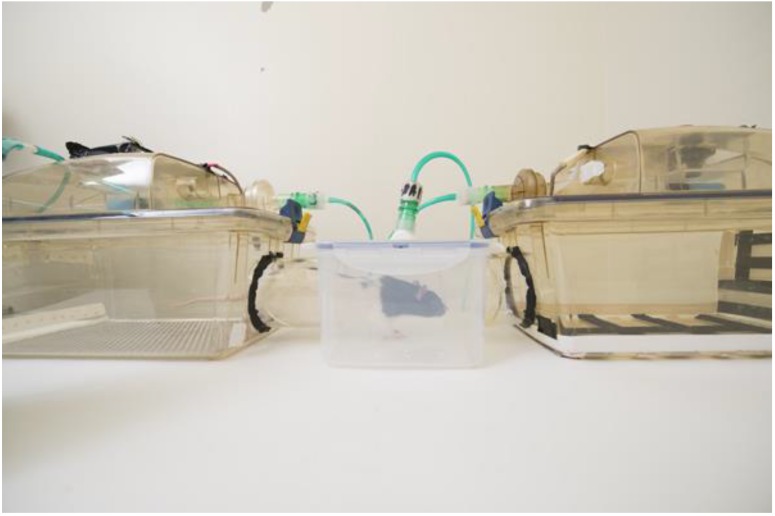
Example of apparatus for conditioned place preference/aversion paradigm. The right and left cage compartments have differently patterned and textured floors and walls to ensure the animal can discriminate between them.

In an unpublished study, Golledge et al. repeatedly exposed male Lister hooded rats to CO_2_ (20% chamber volume per minute flow rate, 2.4 L/min), isoflurane (5%, delivered in 100% oxygen, 2.4 L/min) and argon (chamber prefilled to 98%) to the brink of losing consciousness. These animals had no opportunity to escape from the chamber at any time. The same animals were exposed to air at the same flow rate but in a different chamber. Control and exposure chambers were made distinctive using both visual and tactile cues (Figure 3). Following six conditioning exposures to each of the chambers, the animals were allowed to move freely between the two and their preferences were measured. All three agents caused significant conditioned place aversion. This suggests that rats repeatedly exposed to all three agents have some memory of negative affect during exposure, consistent with the results reviewed earlier showing that repeat exposure is aversive. Golledge called for more research to document the extent of suffering when animals are exposed to different euthanasia agents. This may require the development of and use of more sophisticated testing methods. These findings also underline the need to search for alternative agents for both anaesthesia and euthanasia.

#### 2.1.2. Pain and CO_2_

**Huw Golledge** summarised the evidence from the neuroscience, psychology and physiology literature that exposure to CO_2_, above a certain concentration, is likely to be painful for animals (see [1] for a review). The mechanism for this is well known; carbonic acid is formed when gaseous CO_2_ encounters mucous membranes or other moist tissues. Humans find CO_2_ painful at concentrations over around 50% [23], and rodents have similar **nociceptors** to humans, at a similar density, in their nasal and ocular epithelia. These nociceptors respond to CO_2_ at similar concentrations to human nociceptors [24].

**Figure 4:**
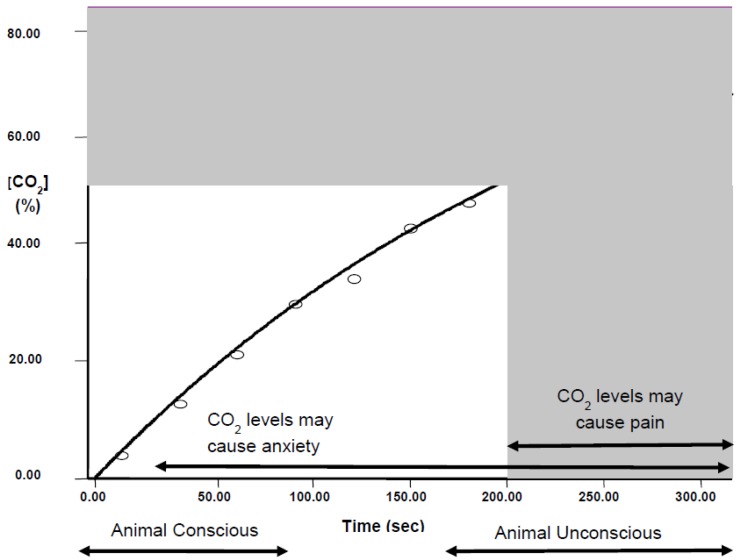
The induction of loss of consciousness during 20% per minute filling with CO_2_. Time line for loss of consciousness in rats exposed to CO_2_ at 20% chamber volume per minute. Animals lose consciousness after approximately 156 s. Bars below the x-axis indicate periods where the animals would be conscious or unconscious. Shaded area shows the likely time/concentration where CO_2_ could cause pain (which occurs after loss consciousness in this case). Levels above 5% may cause anxiety and include a significant duration where the animal would still be conscious.

There are long-standing concerns that CO_2_ killing would be painful for animals [25]; for those placed in a pre-filled chamber this would certainly be the case, but using gradual fill probably avoids causing pain. For example, with a fill rate of 20% chamber volume per minute, rats lose consciousness at 156 ± 5 s (Golledge, unpubl. obs.), at which point the concentration of CO_2_ is 39%, which is likely to be before the gas reaches levels liable to cause pain (Figure 4).

#### 2.1.3. Fear and Anxiety with CO_2_

Carbon dioxide is known to be aversive, at concentrations far below those that cause pain; this aversion is speculated to be due to increased anxiety associated with the inability to escape. There are thus welfare concerns associated with gradual fill because of the long duration when conscious animals are exposed to aversive concentrations of CO_2_ (see Section 2.1.1 above and Figure 4). Mice can detect CO_2_ at near atmospheric concentrations (0.04%) and actively avoid levels of just 0.2% [26], whereas CO_2_ concentrations of up to 30% are odourless to humans [27]. In the mouse, specialised olfactory neurons have been identified that depolarise in response to CO_2_, with a detection threshold of 0.1%. These neurons have **monosynaptic connection** to the **olfactory bulb** in the brain, suggesting a mechanism for CO_2_ detection and avoidance in mice which is not shared with humans [26].

Another potential source of suffering is CO_2_-induced fear, which occurs in both humans and rats. A single breath of 35% CO_2_ can cause panic in susceptible humans, and brief exposure to **normoxic** 20% CO_2_ (The composition of the gas was: 20% CO_2_, 21% O_2_, 59% N_2_) activates “panic/defence” related brain circuits in rats, such as the hypothalamus, rostroventrolateral medulla (RVM) and periaqueductal gray (PAG) [28]. Inhalation of normoxic gas mixtures containing just 7.5% CO_2_ causes anxiety in normal human volunteers if inhaled for longer periods of time [29] and hypervigilance to threat stimuli (e.g., increased orienting towards, and slower orienting away from, the perceived threat) [30]. When inhaling **hypercarbic** gas, people report feeling irritable, nervous, anxious, worried and “feel like leaving”. The anxiety is ameliorated by **anxiolytic** drugs such as benzodiazepines [31].

Carbon dioxide induced **acidosis** also evokes fear responses in mice, such as freezing behaviour which occurs at 5%–10% CO_2_ [32]. Fear responses occur when acidosis is sensed by the ASIC1a channel; genetically altered ASIC1a knockout mice display a significantly reduced fear response to acidosis. Neutralising the pH of the CSF in the **amygdala** also attenuates the fear response.

The potential also exists for CO_2_ to cause **dyspnoea**, anxiety or distress via a third mechanism unrelated to pain. Also referred to as “air hunger”, this is a subjectively uncomfortable and increasingly distressing urge to breathe. Humans may suffer from dyspnoea as a result of asthma or chronic lung diseases. Inhaling CO_2_ can cause dyspnoea in humans [33], and dyspnoea may also occur in rodents (see also [1]).

Golledge argued that, taken together, all of the above strongly suggests that rats and mice exposed to a gradually rising concentration of CO_2_ will experience fear and/or anxiety even if they do not suffer pain. Since the concentrations which cause fear are sub-anaesthetic, all animals killed with CO_2_ are likely to experience these negative states.

**Nicole Marquardt** presented the results of an unpublished study aiming to understand physiological responses during exposure to inhaled agents. Responses were monitored in mice (NMRI and C57/BL6), rats (Wistar and Sprague Dawley) and Syrian hamsters exposed to varying fill rates and concentrations of CO_2_, isoflurane and sevoflurane, with air as a control. The test gases were administered in the animals’ home cages to reduce additional sources of stress (as listed in the introduction to this section). Catecholamine levels were assayed from trunk blood collected by decapitating the animal immediately following the onset of anaesthesia. This study reported some species and strain differences; for example, 8% sevoflurane induced anaesthesia to a surgical plane in 87.5% of NMRI mice but 100% of C57/Bl6 mice, and plasma noradrenaline levels were higher in hamsters compared to rats when they were exposed to 60% CO_2_. In general, the stress hormones adrenaline and noradrenaline were elevated in all mice and hamsters, and most rats exposed to CO_2_, but much less so when rats were exposed to isoflurane or sevoflurane. Thus, these results provide further evidence that CO_2_ causes stress before loss of consciousness, and that volatile anaesthetics are a refinement as they cause reduced induction of stress hormones during the conscious phase of exposure. However, it must be acknowledged that elevation of stress hormones in a conscious animal cannot be conclusively linked to distress, since catecholamine levels have been shown to become elevated in unconscious (anaesthetised) or brain-dead animals [34,35]. Despite this, the agreement of this study with behavioural studies showing stronger aversion to CO_2_ than the other tested agents does suggest that the observed elevations may be related to consciously-experienced negative emotional states.

The above studies, on humans and rodents, indicate that exposure to CO_2_ is likely to cause stress, anxiety and hypervigilance in mice and rats. Other elements of the CO_2_ killing, as commonly conducted, are also likely to be **anxiogenic** (e.g., capture, handling, transport to an unfamiliar environment and mixing with unfamiliar individuals), so CO_2_-induced anxiety will be in addition to that caused by the procedure in many cases. Administering agents in the home cage, or placing established groups in the chamber within their home cage, may minimise some of these stressors, and thus should also be considered a refinement [20]. However, even under “ideal” conditions, CO_2_ is likely to cause acute anxiety.

**Helen Valentine** presented the results of her study comparing physiological and behavioural measures of stress in mice killed with CO_2_—either with or without anxiolytic premedications (acepromazine or midazolam)—to mice anaesthetised with isoflurane. She reported that time to unconsciousness was reduced in midazolam-premedicated animals, but the stress of handling and injecting the mice appeared to negate the benefit of premedication [36]. Corticosterone levels and induction agitation scores were also increased in animals premedicated with midazolam. In addition, midazolam prolonged the time between unconsciousness and death, likely due to its function of increasing the efficiency of the inhibitory transmitter GABA, which has a neuroprotective effect [36]. This will obviously not have welfare implications, but may affect some scientific protocols.

Valentine concluded that signs of stress (both physiological and behavioural) were higher in isoflurane anaesthetised mice than those killed with 20% CO_2_, in contrast to most other studies, including those presented at the meeting by Makowska, Golledge and Marquardt. However, her study and interpretation of the results have been criticised by others—see Makowska et al. [37]. In particular, it has been suggested that inducing anesthesia with isoflurane, then allowing recovery before exposing the animals to CO_2_, has made the results unrepresentative of normal euthanasia procedures where animals would be allowed to become deeply anaesthetised before switching to CO_2_, preventing recovery. The suggestion that mice made distress vocalisations following isoflurane exposure has also been challenged on the basis that there is little evidence of a reliable correlation between ultrasonic vocalisations and painful or aversive stimuli in mice [38], unlike rats. See also Valentine et al. [39] for a response.

#### 2.1.4. Aversion to Anoxia

Low oxygen environments can be created by using an inert gas to displace air from the chamber. Rats and mice are very sensitive to changes in oxygen concentration in inspired air [40]. Makowska reported how, in an approach-avoidance paradigm using argon (one example of an inert gas), rats left the chamber at a mean oxygen concentration of 6.8% and mice at a mean of 8.6% [8,17]. In the aforementioned unpublished study by Golledge et al., a single exposure to 98% argon was found to induce a significant conditioned place aversion, albeit weaker than that observed with repeated conditioning. On the basis of this series of experiments, rats and mice should not be exposed to argon for any reason, including killing.

### 2.2. Physical Methods for Rodents—Decapitation and Cervical Dislocation

There is on-going debate about the relative humaneness of inhalation versus physical methods. Physical methods can be humane within appropriate weight limits, and when effectively performed by trained and competent persons. However, potential welfare concerns and sources of suffering when rodents are killed using decapitation or cervical dislocation include distress from capture and restraint; exposure to other animals’ alarm calls or pheromones; pain or distress experienced between the application of the method and unconsciousness; persistent cortical activity (which potentially indicates on-going consciousness) after application of the method; the possibility of operator error; consequences of operator error; and potential for recovery after incomplete cervical dislocation. Many of these also apply to non-physical methods, but the full list of potential welfare concerns illustrates the greater risk of suffering when physical methods are used.

These concerns are reflected in legislation on the humane killing of laboratory animals. For example, Directive 2010/63/EU [2] permits cervical dislocation for rodents up to 1 kg (requiring sedation for animals weighing over 150 g) or concussion of the brain by striking the cranium for rodents up to 1 kg. The Directive also permits decapitation for rodents “if other methods are not possible”, but this is not a standard method in the UK, where project authorisation is required. Concussion was not discussed within the meeting due to a lack of ongoing research, but the welfare concerns are similar to those for the other physical methods.

The AVMA Guidelines state that decapitation and cervical dislocation are both “acceptable with conditions” for mice and rats (and cervical dislocation only for rats under 200 g) [3]. Conditions include practical factors such as training, operator skill and appropriate equipment, but special justification (e.g., from an ethics or animal care and use committee) for physical methods is not required.

**Larry Carbone** discussed many of the issues surrounding physical techniques, including the failure rate of cervical dislocation, and reviewed some of the science behind these codes of practice and guidelines. With decapitation, the obvious concern is that the decapitated brain may be conscious and in pain. Some studies have made **electroencephalogram** (EEG) recordings in the severed heads of mice and rats, in which animals have been anaesthetised for electrode implantation and then paralysed to allow better quality recordings to be made. A study by Mikeska and Klemm that is 40 years old [41] reported that six paralysed, decapitated rats had persistent “EEG activation” for 5.6–29.5 s (mean 13.6 ± 4.6 SE seconds) and a “total EEG duration” for 19.0–46.5 s. Two other rats had a shift in polarity for over 80 s. Other studies have reported 15–20 s of EEG activity post-decapitation in anaesthetised rats, which was blocked with pre-treatment with atropine [42], while a study on awake rats reported a latency of 17 s to an **isoelectric** EEG [43]. However, whether these results indicate consciousness following decapitation has been debated almost ever since the Mikeska and Klemm study [41]. It should be remembered that EEG can be recorded from animals under surgical anaesthesia and therefore the presence of an active EEG waveform alone is insufficient evidence to conclude that consciousness persists after decapitation [44]. However, the absence of EEG activity or the presence of only a highly suppressed EEG (high amplitude, low frequency activity) does appear to strongly suggest that consciousness has been lost and is commonly accepted to confirm adequate stunning during slaughter of livestock species [45].

On the basis of these studies on decapitated rats, the AVMA [3] urged caution when proposing to kill mice or rats by decapitation *or* cervical dislocation. The AVMA’s recommendations were for special ethics committee or IACUC (Institutional Animal Care and Use Committee) approval and for sedation or “light anaesthesia” before applying the technique.

Cognizant of the uncertainties surrounding older studies of brain neurophysiology in rodents undergoing physical methods of euthanasia, scientists have applied more modern methods. Cartner et al. examined EEG and **visual evoked potentials** (VEP) as evidence of brain electrophysiology during decapitation and cervical dislocation of mice anaesthetised with halothane and paralysed with succinylcholine [46]. With cervical dislocation, there was a significant decrease in VEP after 5–10 s and in EEG after 10–15 s. Durations were longer with decapitation, with a significant decrease in VEP after 10–15 s and EEG after 15–20 s. The times elapsing before responses “flat line” are not given. While this work suggests a rapid progression to unconsciousness, Kongara et al. [44] found that EEG responses observed during the first 10 s following decapitation of anaesthetised rats are consistent with EEG responses to nociception. Taken together, these newer studies suggest that decapitation may lead quite rapidly to unconsciousness, but with conscious pain perception prior to loss of sensation.

With respect to cervical dislocation in rodents, the most recent AVMA guidelines [47] make the point that data suggest electrical activity in the brain persists for 13 s following cervical dislocation in rats, and unlike decapitation, there is no rapid exsanguination to speed the loss of consciousness. The same guidelines also acknowledge that electrical activity in the brain has been demonstrated to persist for 13 to 14 s following decapitation [41], but cite other studies which suggest that such activity does not imply that pain is perceived and which conclude that loss of consciousness is “rapid” post decapitation (e.g., 2.7 s: [48]). VEPs in mice were reduced faster following cervical dislocation than decapitation [46]. Since publication of the AVMA guidelines, a recent report by Kongara et al. [44] found that EEG responses observed during the first 10 s following decapitation of anaesthetised rats are consistent with EEG responses to nociception.

As Carbone argued, the AVMA recommendations assume that cervical dislocation is performed completely efficiently every time, which may be unlikely. There is the potential for non-fatal thoracic and lumbar lesions, so that animals continue to breathe, which indicates that the technique has not been effectively applied and there is a risk that animals may still be conscious. For example, in a study by Carbone et al. [49], mice were anaesthetised with isoflurane and randomised either to one of three cervical dislocation methods: (i) manual, anterograde (in which the operator stabilises the tail with one hand and pushes down and forward on the high cervical region with a twisting motion); (ii) haemostat-assisted; or (iii) intentional thoracic dislocation (a potential accidental outcome of cervical dislocation). Latency to respiratory arrest was measured and post-mortem radiographs and CT scans were used to ascertain where dislocations had occurred. Of the three cervical dislocation techniques, anterograde was the most successful. Even then, however, almost 10% of the mice (2/22) continued to breathe for over 180 s. In pilot studies, some (anaesthetised) mice continued to breathe for over 15 min before being humanely killed. This is a concern because physical damage to the upper spinal cord and brainstem, as would be caused by successful cervical dislocation, should cause rapid respiratory arrest. Whilst ongoing respiration does not indicate consciousness, cessation of breathing induced by damage to the spinal cord and brainstem should occur during cervical dislocation, with concomitant respiratory arrest. The failure to cause respiratory arrest is a sign that the animal has not been rendered brain dead and that there is a possibility of ongoing conscious perception in an injured animal.

This study also indicates that different methods of cervical dislocation should be explored to see whether any are more effective than others. However, the criteria for evaluation should be chosen with care; imaging is not the best predictor of a successful procedure as it is labour-intensive, lesions can be hard to see and results can be difficult to interpret. Anaesthetised mice could be useful for training those required to carry out cervical dislocation (To reduce animal use, animals due to be humanely killed for other reasons should be used wherever possible), using time to respiratory arrest as a measure of success.

It is difficult to apply the data on cervical dislocation to policy on humane killing at present. The few studies conducted to date indicate that brain waves of uncertain significance persist for up to around 15 s and that operator error is a significant risk. In addition, it is as yet unknown how cervical dislocation actually kills animals. The trauma high on the spinal cord leads to respiratory arrest, but there is no significant brain injury. Due to these uncertainties, it is not currently possible to say, from a welfare aspect, how cervical dislocation compares to CO_2_, other inhalants or injectable agents.

The following recommendations can be made, however: more research is needed on the types of operator error that can occur and their consequences; protocols for audits of operator success rates should be developed; equipment should be developed to assist with physical methods of killing (or currently available equipment should be improved as appropriate); and staff should be adequately trained and competent to minimise operator failure.

### 2.3. Euthanasia of Neonatal Rodents

**Craig Johnson** discussed the killing of neonatal animals with respect to their ability to suffer at various developmental stages. Most laws and guidelines that regulate animal experiments do not apply to all “animals”, but rather to those that are assumed to be capable of suffering. In this context, an animal is an organism that has the capacity to suffer because it is of a sufficiently “complex” species and at a relevant developmental stage. Therefore, a noxious stimulus may have a negative impact on this animal’s welfare.

There are different kinds of suffering, but this section of the report focuses on physical pain. In animals older than neonates, this has been commonly measured or inferred using EEG; for example, dehorning in cattle produces marked effects [50]. For a review of neurophysiological techniques that have been used to assess pain in animals, see Murrell and Johnson [51]. The important question is whether and how age may affect the perception of pain, which depends upon the species, its reproductive strategy and developmental characteristics. There is a range of cerebral responses to pain from neonates of very altricial species (e.g., Tammar wallaby [52]), through altricial species (e.g., rats), to precocial species (e.g., sheep [53,54]).

Rats are born moderately neurologically immature, with ear canals and eyes opening between 12 and 14 days postpartum. The EEG is isoelectric for the first 5–7 days, sleeping EEG differentiates between **REM** and non-REM at around 12–18 days, their interest in objects increases by 14 days, and they are play-fighting by 17–20 days. EEG responses to tail clamping in anaesthetised rat pups are moderate at 12–14 days and strong from 21 to 22 days after birth [55]. These results, and the literature, suggest that conscious perception of pain may not be present before 10–12 days old, pups between 12 and 18 days might be capable of some kind of conscious perception, and rats older than 18 days are capable of conscious perception.

The view of Johnson and his colleagues is therefore that the ability of neonatal rodents to suffer whilst undergoing euthanasia is significantly diminished in comparison to later developmental stages. However, the legislation regulating animal use in the European Union [2] assumes that fetal mammals are capable of experiencing suffering during the last third of their development, regulating their use at this stage in the same way as the use of mammals following birth. The UK Animals (Scientific Procedures) Act 1986 also gives developmental stages the “benefit of the doubt”, setting out permitted methods of humane killing for fetal, larval and embryonic forms, including mammals, reptiles and birds [56]. More research is needed into sentience in developmental stages, to better inform decisions about good practice for anaesthesia, analgesia and humane killing.

### 2.4. Euthanasia of Zebrafish

Large numbers of zebrafish (*Danio rerio*) in laboratories worldwide are routinely anaesthetised for scientific and husbandry procedures, and as part of the euthanasia process. Two speakers, **Dan** **Weary** and **Gareth Readman**, presented studies comparing responses of zebrafish to anaesthetic agents. There are very few specific anaesthesia protocols for species of fish (which number over 32,000) and those that do exist are often “historic” rather than properly evaluated for welfare impact and efficacy. The current “standard” anaesthetic used for fish is tricaine methanesulfonate or MS-222 (also known as tricaine mesylate or TMS) ([57]; Figure 2).

Readman described a **chemotaxic** test chamber developed to evaluate aversion of zebrafish to commonly used fish anaesthetics. The chamber was virtually divided into two lanes separated by the creation of a laminar flow within the system, and anaesthetics were introduced via one of two inlets. Agents were then introduced into one half of the tank only, and the fish could choose which half to occupy [58]. This apparatus was used to test the aversive nature of a number of different anaesthetic agents, with the assumption that fish avoid agents they found to be unpleasant.

This study found that zebrafish showed aversion to propoxate, lidocaine hydrochloride, hydrochloric acid (HCl) (positive control), 2-phenoxyethanol (2-PE), MS-222 (buffered to pH 5.0 using sodium bicarbonate), benzocaine, isoeugenol and quinaldine sulphate. The zebrafish showed little or no aversion to etomidate or tribromoethanol (TBE), but TBE can be difficult to store correctly and readily decomposes, forming bromide which is unsafe for staff. The authors concluded that etomidate is an appropriate and practical refinement for euthanasia of adult zebrafish. Incidentally, a cost comparison of the various agents showed that etomidate was relatively inexpensive and cheaper than MS-222.

Weary presented results from a study on conditioned place avoidance responses in zebrafish to common anaesthetics. After only a single exposure to buffered MS-222, all fish showed conditioned place avoidance to MS-222 but not to metomidate or clove oil [59]. These authors concluded that etomidate/metomidate are refinements for euthanasia of adult zebrafish. However, differences between the studies were seen between findings for clove oil and isoeugenol (the active ingredient in clove oil). Wong et al. [59] found no avoidance of clove oil, but Readman [58] found aversion to isoeugenol. These results could suggest a potential system difference caused by the physical/chemical properties of the holding water, or a strain difference within the populations used. Thus further research is required on clove oil and components thereof (NB. Clove oil and its components are classified as rodent carcinogens by the US National Toxicology Program and, because of safety concerns, cannot be used as an anaesthetic in fish in the United States according to the US Food and Drug Administration), and it should also be noted that aversion and safety margins may well vary for other fish species.

Following the presentations and discussion relating to zebrafish, 74% of those participants who voted agreed that they would consider changing the methods they used to kill fish (shown in Figure 2) (17% voted “maybe” and 9% voted “no”). The consensus was that research on fish euthanasia should focus on two main areas; more evaluations of the humaneness of some agents currently in use, and identification of new agents and/or methods, for commonly used species (e.g., methods which are acceptable for tropical species of fish may be problematic for cold water species). A very small number of voters felt that there was no need for further research, and that it had been easier to draw conclusions from the outcomes of research into humane killing of fish than research into euthanising rodents.

### 2.5. Standards of Evidence for Making Decisions about Euthanasia Techniques

Most people require evidence that a new euthanasia technique is “at least as humane” as currently used methods before they will adopt it. This is also a requirement of Directive 2010/63/EU [2]. In addition, many manufacturers of devices for killing animals supply “evidence” that their products are humane. The meeting discussed what kinds of evidence are admissible and how decisions should be made.

**Penny Hawkins** discussed the kinds of factors that could be taken into account when considering humane killing practice at a local level, for example by a researcher wishing to ensure the most appropriate technique is used within her project, or by an ethics or animal care and use committee undertaking a routine review of standard practice at the establishment. Whatever the scenario, a spectrum of “standards” of evidence can be envisaged, as follows, from weakest to strongest: anecdotal evidence/opinion; unpublished data; a single publication; multiple published papers (with compatible conclusions); expert review articles. Usually, multiple confirmatory publications or expert reviews are the minimum that should be used to justify a change in practice. However, sometimes informed judgements can be made if studies appear to provide definitive evidence that could be evaluated within facilities; for example, Readman et al. [58] tested etomidate while Wong et al. [59] tested the analogue metomidate for zebrafish. Given the consistency in the results, despite the differences in agents, testing methods, etc., it seems reasonable to conclude that both agents could be refinements over the existing MS-222 for this species.

Evidence for the humaneness of a euthanasia technique should include factual information to demonstrate that known stressors can be avoided (e.g., capture, handling, restraint, transport and relocation). As success rate is an important factor, data on rates of recovery from the application of the technique should be obtained (e.g., for overdoses), or “death on first attempt”, which is especially important for physical methods from which animals may recover with injuries. Table 1 lists other types of evidence that may be required depending on the protocol.

When deciding between different killing techniques, all of which are compatible with scientific objectives, humaneness (i.e., the animal’s experience) should be the primary consideration. However, if all other factors are equal when choosing between techniques, then it can be justifiable to select a more rapid method (provided that the increased speed does not increase the intensity of pain or distress). “Aesthetics” can be another secondary consideration; some personnel find euthanizing animals psychologically difficult [60] and there is evidence that physical techniques are not preferred by operators [61]. It is important to allow staff to have input into the decision making process with respect to euthanasia techniques for specific projects, but the aim should always be to choose the most humane method.

**Ngaire Dennison** led a discussion concerning the standards of evidence required for regulatory changes to allow new methods for killing certain species. Regulators, such as the UK Home Office, can be faced with some difficult decisions when interpreting and acting upon scientific evidence, especially if results are conflicting or they are expected to extrapolate between different species, strains or developmental stages. The minimum information required to allow a proper consideration of a method is a description of the method; time to final unconsciousness; time to death (both preferably backed up with physiological data); details of ages, sexes and weights for which the method is suitable; how the technique kills; behaviours observed when the method is applied; post mortem evidence of speed/humaneness of death; and how the method compares with other currently permissible protocols. Ideally, this would be published in a refereed journal and reported in accordance with the ARRIVE guidelines ([62], www.nc3rs.org.uk/ARRIVE). Decisions about whether to permit alternative euthanasia techniques are made on the basis of the data provided, whether there is any controversy associated with the method (in which case further information may be needed), and whether there are likely to be any benefits to the animal (e.g., good evidence of lack of distress).

Information on the suitability of the technique with respect to the science is essential for researchers but irrelevant to the consideration of animal welfare. If there is a genuinely justifiable scientific reason for using a killing technique that is not listed as acceptable for general use under the relevant legislation or guidelines, most regulatory systems will allow this provided that a robust scientific case is made, suffering is minimised and the procedure as a whole has successfully undergone a harm-benefit assessment.

Participants were presented with a list of ten types of “scientific evidence” that might be considered acceptable, with the option of voting for as many as they wished. The results are shown in Table 2.

The audience discussed the value of using a combination of both physiological and behavioural evidence when evaluating killing techniques. Behavioural indicators that were regarded as especially important were aversion to an agent “in the moment”; signs of stress or agitation (e.g., escape behaviours); learned or conditioned aversion; and other conditioned responses such as “freezing”. Ultrasonic vocalisations were viewed as less reliable, due to the current lack of ability reliably to interpret these, and the fact that only a limited number of species appear to vocalise as a response to pain or distress.

However, behaviours in the form of movement-related data, such as time to cease moving or **electromyogram** (EMG), rank low in Table 2 because of general awareness that a recumbent animal may still be aware and able to experience suffering. This demonstrates the necessity of using physiological data to either help interpret behaviour, or to use as indicators of awareness and/or suffering if the animal is no longer able to express behaviours. Participants voted for EEG data, levels of stress hormones, heart rate, blood pressure and activation of brain areas associated with negative affective states or stress (e.g., the **HPA axis**) as acceptable evidence to be taken into account when making decisions about killing techniques. It was noted that, although the absence of visual evoked potentials and cortical function indicate that an animal is unconscious, their presence is not necessarily a sign of consciousness, so care is needed when interpreting these data.

Some discussion was also given to the quality of the science, and its interpretation, in studies aiming to evaluate the humaneness of different techniques and protocols. In particular, small sample sizes and some research or reporting methods may miss or mask genuine concerns. For example, in the study of six rats killed by decapitation cited earlier [41], the mean duration of “active” EEG following decapitation was 13.6 s, but the range was 5.6–29.5 s; a five-fold difference. Persistent EEG was reported as a mean of 27.2 s, with a range of 19–465.5 s. There is no such thing as a “mean animal”, and there are clear and serious welfare implications if some animals were experiencing pain for the longer periods. Not only are these outliers important, but a properly powered study could have revealed further insights into the physiology of decapitation and its associated welfare (and ethical) problems. As it was, this study apparently formed the basis for policy, as discussed earlier. It is also worth noting that the mean active EEG has been described as “13–14 s” or “up to half a minute” depending on the viewpoint of the particular author in question.

To conclude, when applying policy or using guidance notes, it is good practice to review the associated references with respect to the experimental design, how long ago the studies were conducted and whether they are still relevant and not superseded by new knowledge, and whether they have been interpreted appropriately and correctly. There is a case for significant care in the analysis and presentation of studies where the outcome is being interpreted with respect to animal welfare; there may be a need for a slight difference from other disciplines as the outlying data represent animals’ experiences that are important, so that a mean or median value is not sufficient to allow consideration of a method’s humaneness.

Participants were presented with a range of suggestions for appropriate mouse and rat euthanasia research directions, which they chose on the basis of the presentations and discussions at the meeting. The majority of votes were for: refinement of existing methods (e.g., home cage euthanasia with inhaled agents); identifying new techniques; gathering evidence as to whether alternatives to CO_2_ (such as isoflurane) might be more humane; and more evidence to inform the debate on the humaneness—or otherwise—of CO_2_.

#### Commercial Devices

Golledge led a discussion on the validation of commercial devices used for killing laboratory animals. “Euthanasia” devices are mainly apparatus for administering inhaled agents or devices for physically killing animals, with a recent upsurge in systems that can administer the agent in the home cage, eliminating stress associated with handling or transport. Many manufacturers have attempted to incorporate recent research findings and recommendations, for example, with respect to fill rates and agents.

Some advantages of various systems, as cited by manufacturers, may relate to animal welfare; for example, home cage administration, automation (reducing the potential for operator error) and reliability. They may also primarily address human safety or economic interests; for example, saving labour (multiple cages or entire racks) and technical support. However, while automation may save resources and obviate some human error, this often comes with reduced human attention towards the animals, which could reduce opportunities to detect and act upon avoidable suffering. From the aspect of a consumer wishing to prioritise animal welfare, these kinds of questions and issues will need to be addressed and an informed judgement made in each case. Buying a particular system could mean a significant outlay and therefore risk being locked into a particular technique, which may become superseded from a welfare aspect.

It will obviously not be possible to tell which device is “most humane” from a list of equivalents on the basis of manufacturers’ literature, nor is it always clear whether claims can be substantiated with robust data. Manufacturers’ literature often uses terms associated with good animal welfare such as “humane” and “kind” with little direct evidence to substantiate these claims. Devices can be tested, but most evaluations of administration techniques are easiest to do using custom built experimental equipment that permits relevant data to be gathered. Manufacturers generally lack the access to animals to directly test their own apparatus, instead relying on end-users to test the apparatus in use.

### 2.6. Input from, and Dialogue with, Ethics or Animal Care and Use Committees

Some legislation and codes of practice suggest, or require, that ethics or animal care and use committees review humane killing techniques that are in use locally. The benefits of input from personnel such as animal technologists and veterinarians may also be emphasised; for example, the U.S. National Research Council Guide states that standardised methods of euthanasia, which are predictable and controllable, should be developed and approved by both the attending veterinarian and IACUC [60]. The institutional Animal Welfare Body required under European Directive 2010/63/EU is also expected to include members with expertise in humane killing, and reviewing euthanasia falls within the Body’s main tasks [63].

The audience was asked whether they had had discussions with ethics or animal care and use committees about killing techniques. Many reported that committees had required justification for a particular technique, had drawn attention to a euthanasia refinement, or had wanted to discuss and review general euthanasia practice at the facility (not in relation to any specific project). Five people reported that a committee had refused to permit a proposed killing technique.

Thirty-four voters responded to a question on how interactions with ethics or animal care and use committees could be improved regarding euthanasia. Of these, 80% agreed with a statement that committee members needed training in understanding the animal welfare and scientific issues; 15% had never had any problems and saw no need for committees to improve; and the remainder felt that such committees should not be commenting on euthanasia techniques.

## 3. Main Points and Areas for Further Research

The list below summarises the topics discussed at the meeting, with the aim of helping to identify areas that require special consideration when making informed decisions on good practice for humanely killing mice, rats and zebrafish.

### 3.1. Inhaled Agents in Mice and Rats

- The majority of participants considered that all inhaled agents tested to date, including CO_2_ and inhaled anaesthetics, are aversive to some degree; animals will avoid exposure when provided with the opportunity.
- Approach-avoidance and aversion-avoidance tests have shown that, in general, isoflurane and sevoflurane are equally aversive to mice and rats, halothane is more aversive than either, and that CO_2_ is inherently more aversive than any of the three other anaesthetics (see Section 2.1.1).
- These tests have also shown that during first exposure to inhaled anaesthetics, more than 50% of rats and mice will choose exposure until they become recumbent rather than abandoning a preferred food reward or crossing over to a brightly lit chamber.
- Results from these tests have shown evidence of *learned* aversion to inhaled anaesthetics; these agents become more aversive after an initial exposure.
- Conditioned place aversion tests, which rely on an animal’s memory of exposure, confirm that repeat exposure to CO_2_, isoflurane and argon cause a negative affective response in rats—demonstrated as a learned aversion to the place where the substances were administered.
- Anoxia (as induced by argon or other gases used to displace air) is highly aversive to rats.

### 3.2. Carbon Dioxide in Mice and Rats

- Mice have specialised olfactory neurons that respond to CO_2_ and, unlike humans, can smell CO_2_ at near-atmospheric concentrations, actively avoiding levels of just 0.2%.
- Exposure to a rising concentration of CO_2_ (e.g., 20% chamber volume per minute) is unlikely to be painful for mice and rats because they lose consciousness before the chamber concentration reaches levels associated with nociceptor activation.
- However, animals exposed to gradual fill as above are still conscious when CO_2_ reaches levels associated with dyspnoea, and anxiety and fear-related behaviours.
- Mice and rats always leave test chambers when the concentration of CO_2_ rises to 12%–18%, even if the cost is to abandon a preferred food reward when they are food-deprived or if the cost is to enter a brightly lit chamber, which is known to be highly aversive.
- Humans breathing normoxic but hypercarbic gas report feelings of fear, anxiety and irritability. In rats, breathing a gas mix of the same composition leads to increased activity in areas of the brain involved in the initiation of fear and anxiety-associated behavioural responses.
- In an evaluation of exposure to CO_2_, plasma levels of the stress hormones adrenaline and noradrenaline (obtained immediately after loss of consciousness) were elevated in mice and rats. Levels were not elevated by the inhaled anaesthetic agents isoflurane or sevoflurane. CO_2_ can elevate catecholamine levels independent of conscious state; however, the agreement of this study with behavioural studies showing stronger aversion to CO_2_ than other tested agents suggests that the observed elevations may be related to consciously-experienced negative emotional states.
- Exposure to CO_2_ is thus likely to cause dyspnoea, anxiety and fear in mice, rats and possibly other rodents. These effects will be exacerbated if the killing process also involves capture, handling, transport to an unfamiliar environment, or mixing with unfamiliar individuals.
- At present, there do not appear to be welfare benefits associated with sedating mice with injectable agents before killing with CO_2_. A study aimed at identifying suitable injectable anxiolytics, such as midazolam, to pre-medicate mice before CO_2_ killing, found that the stress of capture, handling and administering the sedative negated the benefits.
- If mice and rats are anaesthetised using an inhaled agent before switching to CO_2_ to complete the killing process, the change in agent should not be made until they have been recumbent for a sufficiently long period to prevent rapid recovery of consciousness (for mice, evidence indicates waiting a minimum of 80 s after animals become recumbent—see Section 2.1.1).
- This evidence indicates that CO_2_ and agents used to induce anoxia cannot be considered humane methods of euthanasia for rodents, so developing replacements for these agents is an essential goal.

### 3.3. Inhaled Anaesthetic Agents in Mice and Rats

- The majority of evidence shows that the use of halogenated anaesthetics to induce unconsciousness in *naïve* animals is a refinement over the use of CO_2_ or any agent used to induce anoxia.
- When administered using a precision vaporiser, volatile anaesthetics like sevoflurane and isoflurane rapidly induce unconsciousness but may take a long time to cause death in rodents. This makes these agents suitable for inducing unconsciousness (therefore minimising distress during the period within which animals are able to experience poor welfare), but other methods, e.g., cervical dislocation or CO_2_ exposure, can be used to more rapidly kill animals once they have been anaesthetised.
- Rats and mice acquire learned aversion to volatile anaesthetic agents such as isoflurane. During repeat exposure to an inhaled anaesthetic, about half as many animals are willing to tolerate exposure until unconsciousness. This result indicates that any study requiring multiple exposures to anaesthesia (e.g., for surgery or imaging, and later for euthanasia) should be considered high risk.
- Rats “generalise” between different inhaled anaesthetics, so using a different agent for killing will not solve the problem of learned aversion.
- There is a clear need to better understand aversion to inhaled anaesthetics, and in particular the effects of repeated anaesthesia.

### 3.4. Physical Methods for Rodents—Decapitation and Cervical Dislocation

- When performed competently, physical methods may offer a rapid death, which many argue is humane. However, there is on-going debate about the humaneness of some physical methods, including decapitation and cervical dislocation.
- There is concern that a decapitated brain might be conscious and in pain. Both electroencephalogram (EEG) activity and visually evoked potentials (VEP) persist for some seconds in mice and rats, but the significance of this with respect to consciousness and suffering is not yet known. Brain activity also persists following cervical dislocation.
- “Cervical dislocation” can result in lower spinal dislocation and fracture without immediate respiratory arrest, such that animals continue to breathe and may still be conscious.
- In a study of operator success rate for cervical dislocation, the “anterograde” technique (see Section 2.2) proved most effective—but almost 10% of the (anaesthetised) mice continued to breathe for over 3 min, suggesting the technique was not effective.
- Good training, assessment of competence and benchmarking success rates in the use of physical techniques are all paramount with respect to minimising suffering.

### 3.5. Neonates

- The capacity for a neonate to suffer pain depends upon the species and the stage of neurological development at which individuals are born.
- There is evidence that rodents lack the electrophysiological signs believed to relate to conscious brain activity during the first few postnatal days, with consciousness appearing to emerge several days after birth. This suggests that neonatal rodents may lack the ability to suffer pain or distress during this period. However, research is on-going and there is still a case for giving developmental stages the “benefit of the doubt” and treating them as though they can suffer.

### 3.6. Zebrafish

- Avoidance responses and conditioned place-preference tests have been used to evaluate different agents used to kill zebrafish.
- Zebrafish show avoidance of propoxate, lidocaine, hydrochloric acid (HCl), 2-phenoxyethanol (2-PE), buffered MS-222 (also known as tricaine mesylate, tricane methanesulfonate, or TMS), benzocaine, isoeugenol and quinaldine sulphate. They show less evidence of avoidance of etomidate and tribromoethanol (TBE), but TBE is difficult to store and can decompose, forming bromide which is hazardous to humans.
- After just a single exposure to buffered MS-222, zebrafish will consistently avoid the chamber where they had encountered this agent, indicating that this experience was unpleasant for the fish and they remember this experience. Zebrafish show much less evidence of conditioned avoidance of either metomidate or clove oil.
- Together these results indicate that etomidate and metomidate could be humane alternatives for euthanizing zebrafish. Clove oil shows promise in conditioned place aversion testing but avoidance testing shows evidence of aversion to one component (isoeugenol), suggesting that this agent requires more study. The most commonly used agent, MS-222, cannot be considered a humane method for killing zebrafish.

### 3.7. Standards of Evidence for Making Decisions about Techniques for Humane Killing

- Sound scientific evidence is essential when deciding on the most appropriate technique for a particular species and stage of development. Usually this should be in the form of confirmatory publications from multiple laboratories; subjective judgements are not sufficient.
- It is preferable to use a combination of behavioural indicators and physiological data when assessing techniques; for example, a recumbent animal may have an active (high frequency, low amplitude) EEG indicating the possibility of consciousness, or an animal in a state of spasm may have an isoelectric EEG. Supplementary information such as success rates, physicochemical properties of agents or the potential to minimise stressful elements of the process may also be relevant.
- It is good practice to review the references associated with guidelines on humane killing to explore (and question if necessary) the basis for these; as with other areas of science, some guidelines may be based on a small number of studies using few animals or fail to acknowledge the potential implications for animal welfare of data outliers.
- Generalisation between species and strains should be avoided.
- Manufacturers of “euthanasia” devices often attempt to incorporate recent research findings and recommendations, but it is difficult to “test the humaneness” of one against another.

### 3.8. Areas for Further Research

- On-going research into mouse and rat euthanasia should focus on: identifying new techniques; refining current methods (e.g., administration of inhaled agents in the home cage); understanding the cause of learned aversion to inhaled anaesthetics; and assessing the level of suffering caused by different euthanasia agents.
- More studies are required to elucidate the magnitude of aversion in animals exposed to isoflurane more than twice, and the effect on the magnitude of aversion of longer or short intervals between anaesthesia.
- Research should be undertaken to evaluate when consciousness is lost after the various methods, including physical methods. If some level of consciousness persists even after recumbency (for example), there is a potential for severe suffering.
- More research is needed on types and rates of operator error when using various methods of killing such as cervical dislocation and decapitation, with priority given to the consequences for the animal. Protocols for audits of operator success rate should also be developed.
- The possibility that neonatal rodents have some form of consciousness needs to be further investigated.
- Research into fish euthanasia should focus on: (i) evaluations of the humaneness of some agents currently in use and (ii) identifying new agents and/or methods for commonly used species.

## 4. Conclusions

Much progress has been made since the first Newcastle meeting on euthanasia, held in 2006, towards understanding the animal welfare implications of the various killing methods. Some methods are demonstrably less humane than others and should not be used (e.g., all available evidence shows that inducing anoxia using inert gases is more aversive than other inhaled agents for rodents). Some methods are likely only humane when performed correctly (e.g., cervical dislocation) and may cause significant pain and distress if poorly performed. The overwhelming majority of evidence shows that CO_2_ has a negative impact on rodent welfare. Evidence also shows that the volatile anaesthetics provide a potential refinement over both CO_2_ and anoxia for killing rodents. However, some animals show some aversion even to the volatile anaesthetics, suggesting that the search for the ideal inhaled agent(s) should continue. Work on zebrafish has only just begun, but the two studies to date show clear and consistent evidence that etomidate/metomidate is a refinement over the use of MS-222.

On the basis of the meeting and this report, some recommendations for actions at the local, establishment level are:

- Regularly review practice for humane killing, both at the establishment (e.g., via the ethics or animal care and use committee) and, as a researcher, within your own projects.
- Be aware that killing is a process, not an event. It is important to consider the whole experience of the animal, from capture to permanent loss of consciousness.
- When considering humane killing practice, critically review the basis for current guidelines, including the source literature.
- Ensure good communications between researchers and ethics or animal care and use committees, to help ensure that scientific justifications for particular methods are understood by all, and that causes of suffering are recognised and minimised.
- Critically consider device manufacturers’ claims regarding the humaneness of their products, reviewing the literature and seeking expert advice.
- Consider using isoflurane or sevoflurane as a refinement when killing naïve rodents by inducing anaesthesia prior to introducing CO_2_.
- If anaesthetising rodents with isoflurane or sevoflurane before changing to CO_2_, allow sufficient time post-recumbency before changing the agent.
- Do not use injectable anxiolytics before CO_2_ exposure, as this cannot be considered a refinement.
- If it is not possible to change from CO_2_ only in the short term, make sure that the administration protocol reflects current thinking regarding good practice for flow rates, use of diffusers, etc. (see also [1]). Consider administering CO_2_ in the home cage, which will at least eliminate the stress of being moved.
- Never use anoxia (e.g., as induced by argon) to kill rodents.
- Be aware of concerns about physical methods, including both animal and staff welfare. If physical methods are used, ensure that processes are in place to ensure that staff members are trained, competent and willing to use these physical techniques, and that benchmarks for success are set and monitored.
- Do not use decapitation to kill rodents routinely, as this may cause avoidable suffering.
- Follow the literature on sentience in neonatal rodents, giving them the benefit of the doubt with respect to the ability to suffer.
- Use etomidate or metomidate instead of MS-222 to kill zebrafish. Clove oil may also be a suitable refinement to the use of MS-222 but requires more research.
- Keep abreast of the published literature on humane killing techniques.

## Figures and Tables

**Table 1:** Potential adverse effects associated with euthanasia and evidence that can be used to assess these.

Adverse Effect for the Animal	Potential Sources of Suffering or Factors to Consider	Evidence
Pain	Injection Physicochemical properties of agent (e.g., unbuffered PBS, H_2_CO_3_ on mucous membranes) Muscle spasms/seizures Pain from decapitation	Behavioural; physical reactions, vocalisation, attention to site(s) of pain Active EEG Data on duration, incidence, severity of spasms/seizures, observations or **electromyogram** (EMG)
Aversion to inhaled agents	Molecular structure of agent Concentration Flow rate Whether other agents used to induce anaesthesia or as additives Highly species and strain specific	Behavioural; physical reactions (e.g., escape), conditioned place preference/avoidance Information on properties of agent, e.g., pH NOT time to recumbency or to cease movement
Suffering between administration and death	Dyspnoea Pain from injury with physical methods Anxiety, fear Inability to escape from aversive agent Other unpleasant effects of inhaled agents	Behavioural (physical reactions, vocalisation, defecation) Active EEG Respiration rate and depth Corticosterone NOT time to recumbency or to cease movement

**Table 2:** Types of “scientific evidence” that meeting participants considered acceptable for making decisions about the humaneness of a method for killing.

Type of Evidence	Number of Voters
Behavioural indicators of stress, e.g., aversion	47
Behavioural indicators of pain, e.g., attention to eyes or injection sites	47
Positive indicators that suffering is minimal or absent	41
EEG data	40
Success rate	40
Known properties of agents, e.g., pH, mechanism of action	35
Experience of animal before euthanasia process	34
Time to cease moving	28
Time to death	26
EMG and other activity data	19

## Supplementary Materials

The following are available online at www.mdpi.com/2076-2615/6/9/50/s1, Table S1: Meeting Agenda—Second Newcastle Meeting on Laboratory Animal Euthanasia (Newcastle University, United Kingdom, 9 August 2013).

## Glossary

**Acidosis**	an excessively acid condition of the body fluids or tissues.
**Affective states**	emotions and other feelings in animals that are experienced as pleasant or unpleasant.
**Amygdala**	a structure in the limbic system of the brain that is linked to emotions and aggression.
**Anoxia**	absence of oxygen.
**Anxiogenic**	anxiety causing.
**Anxiolytic**	anxiety inhibiting.
**Approach-avoidance test**	a test that uses a desirable reward (e.g., a food treat) to evaluate whether an inhaled agent is aversive and, if so, to what extent. Animals choose whether or not to access the reward in the presence of a range of concentrations of the agent, which provides a way of testing their motivation for the treat against their motivation to avoid the agent. For example, rats and mice always choose to avoid exposure to argon or CO_2_ even when they are food deprived and a preferred treat is present, indicating that these gases are highly aversive.
**Aversion**	avoidance of a stimulus, situation or behaviour.
**Aversive**	causing avoidance of a stimulus, situation or behaviour.
**Aversion-avoidance test**	a test that uses an animal’s motivation to avoid an unpleasant stimulus to evaluate its level of aversion to an inhaled agent. One commonly used paradigm is for rodents to choose between a preferred, dark chamber that is filling with a gas and an aversively brightly-lit chamber. Rats and mice always prefer the bright chamber over one filling with CO_2_, but some choose the dark chamber filling with anaesthetic until they lose consciousness.
**Aversiveness**	see aversion, aversive.
**Chemotaxic**	the movement of an organism in response to a chemical stimulus.
**Conditioned place aversion**	a commonly used technique to evaluate aversion to a specific environment that has been associated with a negative reward.
**Conditioned place preference**	a commonly used technique to evaluate preferences for stimuli that have been associated with a positive reward. In general, this procedure involves several trials where the animal is presented with the positive stimulus (e.g., food) paired with placement in a distinct environment containing various cues (e.g., tactile, visual, and/or olfactory). When later tested in the absence of the stimulus, approaches to and/or the amount of time spent in the compartments previously associated with the positive stimulus serve as indicators of preference.
**Distress**	a strongly negative emotional experience, associated with stress of such magnitude or duration that significant changes in biological function would be necessary for the animal to survive if it could not easily escape the stressor.
**Dyspnoea**	a subjective experience of breathing discomfort; “air hunger”.
**Electroencephalogram (EEG)**	a recording of the electrical activity of the brain, typically representing the activity of the cerebral cortex in mammals.
**Electromyogram (EMG)**	a recording of the electrical activity produced by skeletal muscles.
**Eustress**	“good” or “beneficial” stress, occurring in response to positive events, but which may give rise to the same physiological indicators of stress as negative events.
**Euthanasia**	frequently used to describe the process of killing laboratory animals, euthanasia means a “good death”, which the AVMA [47] defines as a death that occurs with “minimal pain and distress”. The term is also often applied to both humans and animals in the context of ending suffering [64]. Given this, the generic use of the term “euthanasia” for laboratory animals is questionable for two reasons. First, the humaneness of some methods used to kill laboratory animals remains in doubt, such that “killing” is often considered a better description of the processes used to end the lives of laboratory animals. Second, animals are sometimes killed for reasons other than ending their suffering (e.g., if an experiment requires their tissues or if they are surplus to requirements). The term “humane killing” is therefore generally more appropriate than euthanasia in relation to laboratory animals.
**HPA axis**	hypothalamic–pituitary–adrenal axis; the system of feedback interactions among the hypothalamus, pituitary gland, and adrenal glands that controls reactions to stress and regulates many body processes.
**Humane endpoint**	one or more predetermined physiological or behavioural signs that define the point at which an experimental animal’s pain and/or distress will be terminated, minimized or reduced by taking actions such as killing the animal, terminating a painful procedure or giving treatment to relieve pain.
**Humaneness**	characterised by concern or compassion for others; in the context of methods of humane killing, that which causes little or no pain or distress.
**Hypercarbic**	containing excess CO_2_.
**Hypoxia**	deficiency in the amount of oxygen reaching the tissues.
**Inhaled agent**	a substance (gas or vapour) which is administered via the inhalation route.
**Isoelectric**	having or involving no net electric charge or difference in electrical potential; in EEG a signal representing no on-going brain activity.
**Monosynaptic connections**	passing through a single synapse (junction between two nerve cells).
**Neonatal**	relating to the immediate period after birth. The age of neonatal rodents has been defined as up to and including day 10, where the day of birth is day 1 [56].
**Nociceptor**	a sensory receptor (nerve) for painful stimuli.
**Normoxic**	containing the normal atmospheric concentration of oxygen (~21%).
**Olfactory bulb**	a neural structure of the vertebrate forebrain involved in olfaction or the sense of smell.
**Pavlovian association**	an association between a previously unrelated neutral stimulus (e.g., bell) and another stimulus (e.g., food) that reliably elicits a reaction (e.g., salivation).
**REM**	REM (rapid eye movement) sleep.
**Visually evoked potential (VEP)**	electrophysiological responses to stimulation by either patterned or unpatterned visual stimuli.
