# Supplementary material for: A Good Death? Report of the Second Newcastle Meeting on Laboratory Animal Euthanasia
Source: Animals (Basel). 2016 Aug 23;6(9):50. doi: 10.3390/ani6090050 (PMC5035945; doi:10.3390/ani6090050)
Supplement: Supplementary file 1 [file animals-06-00050-s001.pdf]

Supplementary Materials: A Good Death? Report of the Second Newcastle Meeting on Laboratory Animal Euthanasia

Penny Hawkins, Mark J. Prescott, Larry Carbone, Ngaire Dennison, Craig Johnson, I. Joanna Makowska, Nicole Marquardt, Gareth Readman, Daniel M. Weary and Huw D. R. Golledge

Table S1. Meeting Agenda—Second Newcastle Meeting on Laboratory Animal Euthanasia (Newcastle University, United Kingdom, 9 August 2013).

Time	Programme
08:30–09:00	Registration
09:00–09:05	Introductory remarks
09:05–09:25	Introduction and discussion<br>*Huw Golledge, Newcastle University, Penny Hawkins, RSPCA and Mark Prescott, NC3Rs*
09:25–09:45	Rodent aversion to CO_2, isoflurane and argon<br>*Joanna Makowska, Animal Welfare Program, University of British Columbia*
09:45–10:05	Conditioned place aversion to inhaled euthanasia agents in rats<br>*Huw Golledge, Centre for Behaviour and Evolution, Newcastle University*
10:05–10:25	Sedation or inhalant anaesthesia before euthanasia with CO_2 does not reduce behavioural or physiologic signs of pain and stress in mice.<br>*Helen Valentine, Office of Animal Resources, University of Iowa*
10:25–10:45	A multi-layered approach comparing the euthanasia agents CO_2, isoflurane and sevoflurane in mice, rats and hamsters<br>*Nicole Marquardt, Institute of Pharmacology and Toxicology, Department of Veterinary Medicine, Freie Universität Berlin*
10:45–11:15	Coffee break
11:15–11:35	Carbon dioxide—reasons to be fearful<br>*Huw Golledge, Centre for Behaviour and Evolution, Newcastle University*
11:35–11:55	Physical euthanasia techniques for rodents<br>*Larry Carbone, Lab Animal Resource Center, University of California San Francisco*
11:55–12:15	Euthanasia of neonatal rodents – are they conscious and can they suffer?<br>*Craig Johnson, Institute of Veterinary Animal and Biomedical Sciences, Massey University*
12:15–13:00	Discussion: Rodent euthanasia<br>*Moderators: Penny Hawkins, RPSCA and Mark Prescott, NC3Rs*
13:00–14:00	Lunch
14:00–14:10	Conditioned place preference responses of zebrafish to tricaine methanesulfonate, metomidate hydrochloride, and clove oil<br>*Devina Wong, Marina von Keyserlingk, Jeffrey Richards and Daniel Weary, Animal Welfare Program, University of British Columbia*
14:10–14:25	Fish anaesthesia – are commonly used anaesthetics aversive?<br>*Gareth Readman[1,2], Toby Knowles[2], Stewart Owen[1] and Jo Murrell[2], [1] School of Veterinary Science, University of Bristol; [2] Brixham Environmental Laboratory, AstraZeneca*
14:25–14:45	Discussion: Commercial devices for killing lab animals – what's available and how should we validate them?<br>*Moderator: Huw Golledge, Newcastle University*
14:45–14:55	Why are outliers important?<br>*Larry Carbone, Lab Animal Resource Center, University of California San Francisco*
14:55–15:05	Do we care about the final time an animal loses consciousness more than any other?<br>*Bryan Vernon, Senior Lecturer in Medical Ethics, Medical School, Newcastle University*
15:05–15:30	Coffee break
15:30–16:00	What standards of evidence should we rely on to decide whether euthanasia techniques are humane?<br>*Ngaire Dennison, Animals (Scientific Procedures) Inspectorate, Home Office and Penny Hawkins, RSPCA*
16:00–17:30	Discussion<br>*Moderators: Penny Hawkins, RSPCA and Mark Prescott, NC3Rs*
17:30	Meeting close

© 2016 by the authors. Submitted for possible open access publication under the terms and conditions of the Creative Commons Attribution (CC-BY) license (http://creativecommons.org/licenses/by/4.0/).
